# Lipid peroxidation is not associated with adipocytokines in preeclamptic women

**Published:** 2011

**Authors:** Ali Khosrowbeygi, Nahid Lorzadeh, Hassan Ahmadvand

**Affiliations:** 1 Department of Biochemistry, School of Medicine, Lorestan University of Medical Sciences, Khorramabad, Iran.; 2 Department of Obstetrics and Gynecology, School of Medicine, Lorestan University of Medical Sciences, Khorramabad, Iran.

**Keywords:** *Lipid peroxidation*, *8-Isoprostane*, *Malondialdehyde*, *Leptin*, *Adiponectin*, *Preeclampsia*, *Pregnancy*

## Abstract

**Background:** The association between lipid peroxidation biomarkers and adipocytokines is hypothesized in preeclampsia but little is known about it.

**Objective:** he present study was undertaken to determine the association of lipid peroxidation biomarkers measured by the levels of 8-isoprostane and malondialdehyde with adipocytokines leptin and adiponectin in maternal serum of preeclamptic women.

**Materials and Methods:** A cross-sectional study was designed. The study population consisted of 30 preeclamptic patients and 30 healthy pregnant women. Serum levels of 8-Isoprostane, total leptin and total adiponectin were assessed using commercially available enzyme-linked immunosorbent assay methods. The amount of malondialdehyde was determined by the thiobarbituric acid assay. Mann-Whitney U test and Spearman’s correlation coefficient were used for statistical analysis.

**Results:** Both 8-Isoprostane and malondialdehyde were significantly higher in preeclamptic group than those in normal pregnant women. Serum levels of leptin were significantly increased in preeclamptic patients compared with normal group, while adiponectin levels were decreased. Serum levels of 8-Isoprostane and malondialdehyde did not show any significant correlation with leptin and adiponectin.

**Conclusion:** In preeclamptic women, maternal serum levels of leptin and adiponectin were not correlated with lipid peroxidation.

## Introduction

Preeclampsia, a pregnancy specific syndrome, is a major cause of maternal and perinatal morbidity and mortality (1). Preeclampsia is characterized by the onset of hypertension and proteinuria after 20 weeks of gestation in a previously normotensive pregnant woman (2-4). The exact mechanism underlying etiology of preeclampsia remains elusive (1, 5, 6). There are many theories about the etiology and pathogenesis of preeclampsia including the presence of inflammation and angiogenesis (7). 

 It has been proposed that excess oxidative stress and exaggeration of a maternal inflammatory response are involved in the pathogenesis of preeclampsia (6, 8-11). Leptin is an adipocytokine that is secreted primarily by white adipose tissue. It is also produced by human placental trophoblasts (2, 3, 7, 8). Leptin stimulates inflammatory responses via induction of proinflammatory cytokines in placenta and adipose tissue (6). Adiponectin, a specific adipocyte derived hormone, has been considered to improve insulin sensitivity, inhibit vascular inflammation and atherogenesis (7).

It was shown that leptin causes lipid peroxidation in cultured human endothelial cells, therefore it has been suggested that leptin promotes atherogenesis in preeclampsia (12). Based on the assumption that there are interactions in the effects of oxidative stress and adipocytokines on vascular endothelial cells and consequently in the development of preeclampsia, it has been hypothesized that lipid peroxidation biomarkers are associated with adipocytokines in the disease (6, 7). Little is known about the association between maternal serum levels of lipid peroxidation biomarkers and adipocytokines in preeclamptic patients. It was shown that in Chinese preeclamptic women there is no significant correlation between plasma levels of adipocytokines and 8-Isoprostane (6). Therefore, a cross-sectional study was designed to determine the association between 8-Isoprostane and malondialdehyde, as lipid peroxidation biomarkers, and adipocytokines leptin and adiponectin in preeclamptic women compared with normal pregnant women.

## Materials and methods

**Subjects**

A cross-sectional study was designed. The study was approved by Institutional Ethical Review Board, and informed consent was obtained from each pregnant woman enrolled in this study. Sixty pregnant women were included in the present study. Of these, 30 women were preeclamptic patients and 30 age-, gestational week-, and body mass index (BMI)-matched were as normal group. Preeclampsia was defined as blood pressure equal to or higher than 140/90 mmHg with proteinuria of either higher than 100 mg/dl by urine analysis or higher than 300 mg in a 24-h urine collection. Severe preeclampsia was defined as blood pressure equal to or higher than 160/110 mmHg (2). 

Exclusion criteria were smoking, multiple gestation, diabetes mellitus, chronic hypertension, heart failure, inflammatory or infective disorders, and infectious disease (7). Blood samples were collected from preeclamptic patients at the time of acceptance to the hospital shortly after the preeclampsia diagnosis was confirmed. Serum samples were stored at -70°C until examination.

**8-Isoprostane assessment**

We assessed free form of 8-Isoprostane. “At first, free 8-Isoprostane was purified by affinity chromatography method. We used commercially available affinity column (Cayman Chemical, Ann Arbor, MI, USA). All samples were centrifuged briefly at 1500 rpm for isolating of particulates and precipitates. Then the supernatant was diluted 1:5 with column buffer and applied to the column. Other procedures were according to the instructions provided by the manufacturer. The ethanol washed 8-Isoprostane stored at -20°C until measurement. For measuring 8-Isoprostane, the elution solution was evaporated to dryness under a stream of nitrogen gas. The dried samples were dissolved in buffer. Then, the concentration of free 8-Isoprostane was measured by enzyme immunoassay (EIA) method. We used commercially available EIA method (Cayman Chemical, Ann Arbor, MI, USA). The procedure for the EIA was according to the instructions provided by the manufacturer. The sample volume that used was 50 μl. Absorbance was measured at a wavelength of 405 nm using enzyme-linked immunosorbent assay (ELISA) reader (STAT FAX 2100, USA) and levels of 8-Isoprostane were presented as pg/ml. The intra-assay coefficient of variation was <10%. The detection limit and specificity of 8-Isoprostane assay were 5 pg/ml and 100%, respectively” (13). 

**Malondialdehyde measurement**

The amount of malondialdehyde (MDA) was determined by the thiobarbituric acid (TBA) assay (14). All reagents were obtained from Merck Company (Darmstadt, Germany). Briefly, 1 vol of serum was added to 6 vol of phosphoric acid [1.00% (w/v)] in 0.1 N HCl, 2 vol of TBA [0.60% (w/v)] in 0.1 N HCl. The samples were heated in a boiling water bath for 45 minutes. After cooling the chromogen was extracted in 8 vol of n- butanol. The absorbance of the organic phase was measured at a wavelength of 532 nm. The concentration of MDA was expressed as μmol/l using a molar absorption co-efficient of 156000 M^-1^cm^-1^.

**Leptin and adiponectin** **measurement**

The concentrations of total leptin and adiponectin were measured using ELISA method. We used commercially available human leptin and adiponectin ELISA methods (BioVendor Laboratory Medicine Inc. Czech Republic). The procedures for the methods were according to the instructions provided by the manufacturer. The sample volumes that used were 50 µl in the leptin assay and 10 µl in the adiponectin assay. Absorbances were measured at a wavelength of 405 nm using ELISA reader (STAT FAX 2100, USA). The levels of leptin and adiponectin were presented as ng/ml and µg/ml, respectively. The intra-assay coefficients of variations of the methods were <10%. The sensitivity and specificity of the leptin assay were 0.2 ng/ml and 100%, respectively. The sensitivity and specificity of the adiponectin assay were 26 ng/ml and 100%, respectively (BioVendor Laboratory Medicine Inc. Czech Republic).

**Statistical analysis**

“Based on a literature review, using α value of 0.05 and a β value of 0.2 (80% power), the minimum sample size required was calculated 30 samples per group” (13). Sample size was calculated using the formula for two independent samples (15). “Differences between case and control groups were assessed using Mann-Whitney U test. Coefficients of correlation were calculated using Spearman’s correlation analysis. All hypothesis tests were two-tailed with statistical significance assessed at the p-value<0.05 level with 95% confidence intervals” (14). The levels of leptin and adiponectin were adjusted for BMI (1). “The data are expressed as the Mean±SEM. Statistical computations were calculated using SPSS 11.5 for windows software (SPSS Inc, Chicago, IL, USA)” (13).

## Results

haracteristics of normal pregnant women and preeclamptic patients are shown in Table I. There were no significant differences in age, gestational age, and BMI between preeclamptic and normal pregnant women. Systolic and diastolic blood pressures were significantly higher in preeclamptic women than those in healthy pregnant women. Serum levels of lipid peroxidation biomarkers and adipocytokines in normal pregnant women and preeclamptic patients are shown in Table II. Both 8-Isoprostane and malondialdehyde were significantly higher in preeclamptic group than those in normal pregnant women. Compared with normal group, serum levels of leptin were significantly increased in preeclamptic group, while adiponectin levels were decreased.

Patients were then stratified to mild (n=17) and severe (n=13) preeclampsia. There was no significant difference in 8-Isoprostane levels between mild and severe preeclampsia data not shown). Women with severe preeclampsia had significantly higher serum levels of malondialdehyde than mild preeclampsia (6.36±0.42 vs. 3.97±0.14 μmol/l, p=0.000)data not shown).

**Figure 1:**
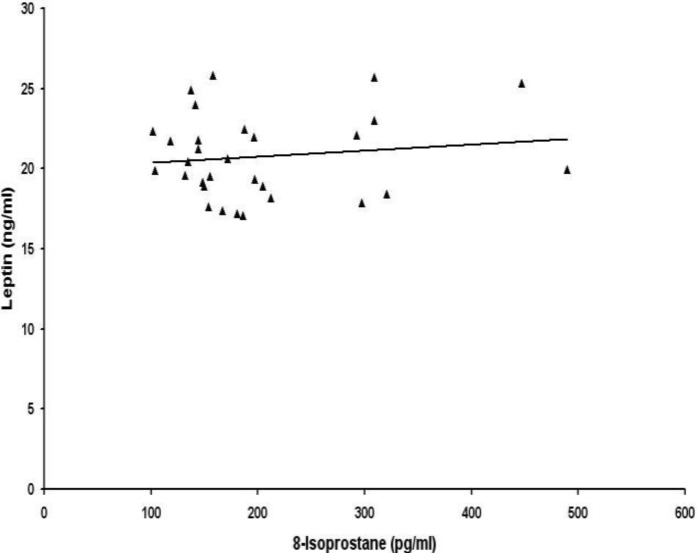
Correlation between maternal serum levels of 8-isoprostane and total leptin in women with preeclampsia (r = 0.138, p>0.05).

**Figure 2:**
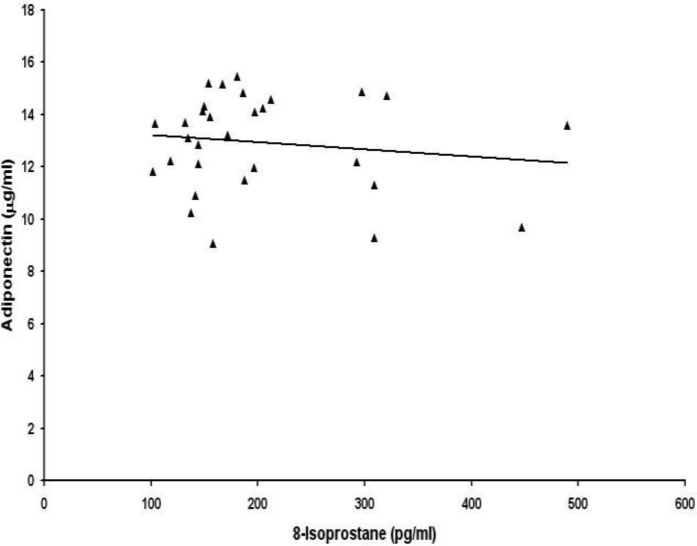
Correlation between maternal serum levels of 8-isoprostane and total adiponectin in women with preeclampsia (r=-0.141, p>0.05).

**Figure 3:**
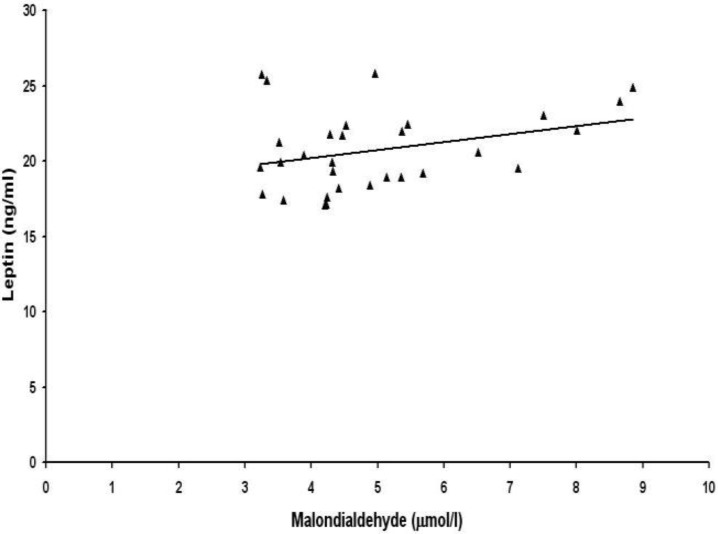
Correlation between maternal serum levels of malondialdehyde and total leptin in women with preeclampsia (r=0.286, p>0.05).

**Figure 4:**
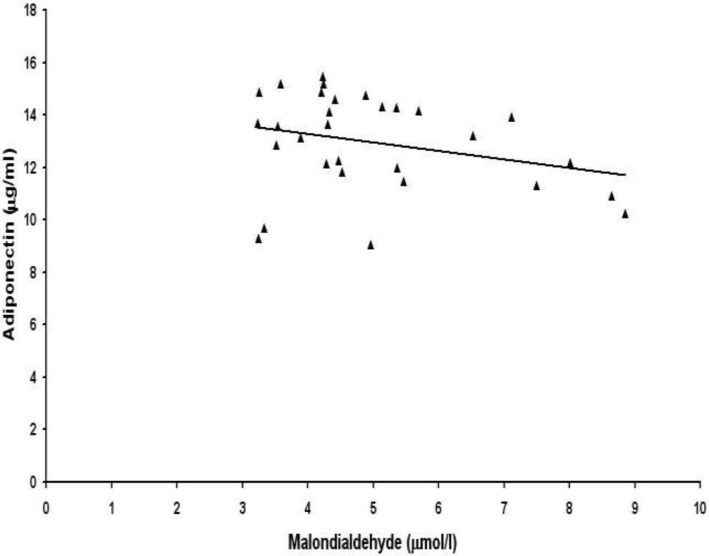
Correlation between maternal serum levels of malondialdehyde and total adiponectin in women with preeclampsia (r = -0.269, p>0.05).

Then we examined the correlations between lipid peroxidation biomarkers and adipocytokines leptin and adiponectin. Serum levels of 8-isoprostane were not correlated with leptin (Figure 1) and adiponectin (Figure 2) in preeclamptic women. No association was found between serum levels of malondialdehyde and adipocytokines leptin (Figure 3) and adiponectin (Figure 4) in women with preeclampsia. No correlation was observed between serum levels of lipid peroxidation biomarkers and adipocytokines in normal pregnant womendata not shown). There was a significant negative correlation between leptin and adiponectin in normal pregnant women (r=-0.98, p=0.000) and also in preeclamptic patients (r= -0.99, p= 0.000)data not shown)

Levels of malondialdehyde correlated positively with systolic blood pressure values in preeclamptic women (r= 0.42, p= 0.001)while 8-Isoprostane had no correlation data not shown). When both preeclamptic and normal pregnant women were pooled, levels of 8-Isoprostane showed a significant correlation with systolic (r=0.42, p= 0.001) and diastolic (r= 0.71, p= 0.000) blood pressures data not shown).

**Table I:** Characteristics of preeclamtic and normal pregnant women

**Parameter**	**Normal** ** pregnant (n=30)**	**Preeclamptic (n=30)**	**p-value**
Age (years)	31.07 ± 0.35	32.27 ± 0.69	0.2
Gestational age of blood sampling (weeks)	39.10 ± 0.23	38.50 ± 0.24	0.09
Third trimester BMI (kg/m^2^)	26.23 ± 0.51	27.88 ± 0.78	0.1
Systolic blood pressure (mm Hg)	109.00 ± 1.11	150.67 ± 1.91	0.001
Diastolic blood pressure (mm Hg)	66.33 ± 1.31	95.67 ± 2.23	0.001

Results are presented as mean ± SEM. BMI, body mass index.

**Table II:** Maternal serum levels of 8-Isoprostane, malodialdehyde (MDA), leptin and adiponectin in preeclamtic and normal pregnant women

**Parameter**	**Normal** ** pregnant (n=30)**	**Preeclamptic (n=30)**	**p-value**
8-Isoprostane (pg/ml)	37.06 ± 2.77	202.97 ± 17.39	0.00
MDA (μmol/l)	3.81 ± 0.48	5.00 ± 0.29	0.03
Leptin (ng/ml)	19.69 ± 0.28	20.75 ± 0.48	0.04
Adiponectin (µg/ml)	14.00 ± 0.22	12.94 ± 0.34	0.01

Results are presented as mean ± SEM.

## Discussion

The most relevant finding of this study is that no significant correlation was observed between lipid peroxidation and adipocytokines in preeclamptic women. Our findings were in line with the studies reporting the increased lipid peroxidation assessed as circulating levels of 8-Isoprostane in preeclamptic patients compared with normal pregnant women (6, 16). However, our data were not agreed with Ishihara *et al* (17) study that showed there is no significant difference in 8-Isoprostane levels between preeclampsia and normal pregnancy. 

In the present study, maternal serum levels of malondialdehyde were significantly increased in preeclamptic patients compared with normal pregnant women. This finding was in concordance with earlier reports (18-26). However, our finding was not agreed with other studies reporting there is no significant difference in circulating levels of malondialdehyde between preeclamptic and normal pregnant women (27, 28).

Our data confirm the results obtained by others (2, 6, 7, 11, 29), who observed that circulating levels of leptin are significantly higher in preeclamptic patients than in normal pregnant women. 

“Isoprostanes are formed in situ in cell membranes; following free radical attack on the arachidonic acid. Unlike prostaglandins, which are formed from arachidonic acid following its release from the *sn*-2 position of phospholipids by phospholipase A2, isoprostanesare formed initially in situ, where they may contribute to the effects of oxidative stress on membrane biophysics. Measurement of 8-isoprostane may provide a reliable marker of lipid peroxidation in vivo, because, it is a stable compound. In addition, 8-Isoprostane is specific product of free radical-induced lipid peroxidation. 8-Isoprostanehas also has been found to be present in detectable quantities in all normal biological tissues and in free form in all normal biological fluids. This is important because it allows the definition of a normal range such that small increases in its formation can be detected in situations of mild oxidative stress. Finally, the levels of 8-isoprostane is unaffected by lipid content of the diet” (13).

Hemoconentration is a main characteristic of preeclampsia. Therefore, it could be considered that increasing in leptin and decreasing in adiponectin levels is due to hemoconentration present in this syndrome. However, it was shown that significant difference in levels of adipocytokines between preeclamptic and normal pregnant women is not affected by correcting adipocytokines levels for hematocrit (29). 

“Although oxidative stress is implicated in the pathogenesis of preeclampsia, it remains unclear whether it is a primary or secondary mediator of increased blood pressure” (30). It has been reported that leptin causes oxidative stress in cultured human endothelial cells (12). Therefore, it may be assumed that oxidative stress would be associated with leptin in preeclampsia (6). In the present study, no correlation was observed between serum levels of leptin and lipid peroxidation biomarkers. This finding confirmed the results obtained by Ouyang *et al* (6), who hypothesized that excess oxidative stress resulting from placental hypoxia could be an early event in the pathogenesis of preeclampsia, causing placental endothelial damages. It has been hypothesized that hyperleptinemia might contribute to the decreased production of adiponectin by adipocytes (7). In our study, there was a significant negative correlation between leptin and adiponectin in both preeclamptic and normal pregnant women that might confirm the hypothesis. Although leptin causes oxidative stress in cultured human endothelial cells, no association was found between serum levels of leptin and oxidative stress biomarkers in previous (6) and the present studies. Therefore, the findings of the present study could support the hypothesis that oxidative stress is primary mediator in the progression of preeclampsia, contributing to endothelial damage in the placenta (6, 30). We can conclude that in preeclamptic women, serum levels of leptin and adiponectin were not correlated with lipid peroxidation.
